# Predicting survival time for metastatic castration resistant prostate cancer: An iterative imputation approach

**DOI:** 10.12688/f1000research.8628.1

**Published:** 2016-11-16

**Authors:** Detian Deng, Yu Du, Zhicheng Ji, Karthik Rao, Zhenke Wu, Yuxin Zhu, R. Yates Coley

**Affiliations:** 1 Department of Biostatistics, Johns Hopkins University, Baltimore, USA; 2 School of Medicine, Johns Hopkins University, Baltimore, USA

**Keywords:** Iterative imputation, multiple imputation, Ensemble learning, Survival Time Prediction

## Abstract

In this paper, we present our winning method for survival time prediction in the 2015 Prostate Cancer DREAM Challenge, a recent crowdsourced competition focused on risk and survival time predictions for patients with metastatic castration-resistant prostate cancer (mCRPC). We are interested in using a patient's covariates to predict his or her time until death after initiating standard therapy. We propose an iterative algorithm to multiply impute right-censored survival times and use ensemble learning methods to characterize the dependence of these imputed survival times on possibly many covariates. We show that by iterating over imputation and ensemble learning steps, we guide imputation with patient covariates and, subsequently, optimize the accuracy of survival time prediction. This method is generally applicable to time-to-event prediction problems in the presence of right-censoring. We demonstrate the proposed method's performance with training and validation results from the DREAM Challenge and compare its accuracy with existing methods.

## 1 Introduction

Predicting overall survival for cancer patients remains central to studying new treatment options. Given a patient’s covariates and preferences, doctors can anticipate prognosis and likely treatment effects and make clinical recommendations accordingly. For example, docetaxel is a standard treatment for patients with metastatic prostate cancer who have developed resistance to conventional androgen deprivation therapy. Using data from the docetaxel arm of four recent phase III trials of experimental interventions, the 2015 Prostate Cancer DREAM Challenge ^1^ aims to amass community-based efforts to develop, apply, and validate prognostic models for overall patient survival under this standard treatment.

A frequently encountered problem in survival analysis is data censoring, in which exact survival times are not observed for all patients. The most common type of censoring is right censoring, in which the survival time is only observed up to a certain censoring time; event times are not observed for individuals after censoring occurs. Many state-of-the-art statistical and machine learning tools cannot be directly applied to censored data while most standard methodologies that do allow for censoring assume independence between censoring and survival time; this assumption is frequently inappropriate.

Among survival analysis methods that accommodate censoring, many approaches focus on maximizing the partial likelihood, which depends only on the order of events rather than the time at which they occur. One of the most widely used methods, the proportional hazards model (also known as the Cox regression model) parameterizes this partial likelihood through a baseline hazard function and a multiplicative scaling term that depends on covariates ^2, 3^. Other methods in this class often seek different formulations of the hazard function. For instance, proportional hazard models based on artificial neural networks ^4, 5^ and the gradient boosting proportional hazard model ^6^ have been developed to model more complex forms of the non-linear hazard function.

Alternate objective functions have also been developed for survival analysis with censored data. Support vector regression techniques can be adapted to survival time prediction by considering censored outcomes as interval targets and forming a new maximum margin loss function directly with log-transformed survival time ^7^. In random survival forests (RSF) ^8, 9^, a tree-based ensemble model that relies on bagging, each survival tree split is determined by maximizing the survival difference ^10^ between child nodes. More recently, a gradient boosting-based model with direct optimization of Harrell’s concordance index has been developed ^11, 12^.

As an alternative to the above methods that directly accommodate right-censored survival data, multiple imputation ^13^ methods treat the censored observations as missing data. To overcome the obstacle posed by censoring, these methods randomly generate missing survival outcomes many times in order to permit complete-data inferences. Taylor *et al*.(2002) ^14^ propose two nonparametric imputation methods that enable estimation of the survival distribution for right-censored survival data without covariates. One approach, risk set imputation (RSI), replaces an individual’s censored time with a random draw of observed event times among those at risk (beyond the particular censoring time), starting from the smallest and proceeding toward the largest censored time. With an infinite number of imputations, RSI survival point estimates are equivalent to the Kaplan-Meier estimator, $E\{{\hat{S}}_{RSI}(t)\}={\hat{S}}_{KM}(t),$ where the expectation is taken with respect to the distribution of all possible random imputations. This imputation technique does not use the covariate data which, if modeled jointly with survival times, can improve accuracy of survival time predictions.

Conditional survival estimates are more informative for individual survival time predictions. Unbiased conditional survival estimation, i.e., $E\{{\hat{S}}_{RSI}(t;x)\}={\hat{S}}_{KM}(t;x)$ ensures unbiased population-averaged survival curve estimation, $E\{{\hat{S}}_{RSI}(t)\}={\hat{S}}_{KM}(t)=E_{X}[{\hat{S}}_{KM}(t;x)],$ while the reverse does not hold. Given a covariate-specific survival distribution estimate, $\hat{\mathrm{Pr}}(T_{i}>t\text{|}X_{i}),$ ∀ *t* > 0, it remains open as to how to predict an individual’s exact survival time ( *T _i_*). Our method approaches this problem from another perspective by directly modeling survival times.

In this paper, we propose a new method for exact survival time prediction that relies on strategically imputing censored time and, then, building an ensemble prediction model based on the “complete” dataset. In so doing, we are able to exploit the predictive power of many state-of-the-art regression technologies. This imputation algorithm first multiply imputes censored survival times in order to construct a complete dataset without using covariates. Then, the algorithm iterates between 1) predicting the completed survival times using covariates and 2) adjusting the imputed value.

In the following, we first describe the data for training, testing, and validating our proposed survival time prediction model and, then, summarize the statistical methods that we used to construct the ensemble model. We conclude by discussing potential directions for future research and further improvements.

## 2 Data

Data from the control arm of four phase III clinical trials of experimental therapies for mCRP were made available to participants in the Prostate Cancer DREAM Challenge. The trials are ASCENT-2 (conducted by Memorial Sloan Kettering Cancer Center) ^15^, VENICE (Sanofi) ^16^, MAINSAIL (Celgene) ^17^, and ENTHUSE-33 (AstraZeneca) ^18^. Training data include survival outcomes (time of death or censored survival time) and 131 clinical covariates from the ASCENT-2, MAINSAIL, and VENICE trials. Only covariate data were available for the ENTHUSE-33 trial; survival outcomes were blinded for scoring. Clinical covariates included patient demographics, vital signs, lab results, medical history, medication use, and tumor measurements.

### 2.1 Data cleaning and summaries

***2.1.1 Data consolidation***: A primary dataset, referred to here as the “CoreTable", was provided by the DREAM Challenge organizers and summarized many relevant baseline covariates at patient level. An additional five raw datasets containing more detailed baseline and follow-up data were also provided. We summarized additional baseline information from these secondary tables to augment the CoreTable. For example, medications were grouped according to drug type or use including opiod analgesics, anti-depressants, and vitamin supplements. Tumor data were also summarized across disease sites including the number, average size, and maximum size of lesions. Continuous lab values were log-transformed; non-transformed values were also kept in the data. Covariate data from secondary tables that duplicated or were highly correlated with existing variables in the CoreTable were excluded from the analysis.

The resulting dataset had 2070 observations and 256 covariates, among which 78 covariates were continuous variables and 177 were categorical variables.

***2.1.2 Splitting data for 10-fold cross-validation***: In order to maintain consistent groupings for cross validation, we evenly split the training data into ten groups by randomly generating a uniform 10-fold index for each observation. As a result, we were able to maintain the same hold-out datasets as we employed different prediction methods. When generating the random 10-fold index, we set a random number generation seed for reproducibility (DOI: 10.7303/syn4732982).

**Figure 1.**
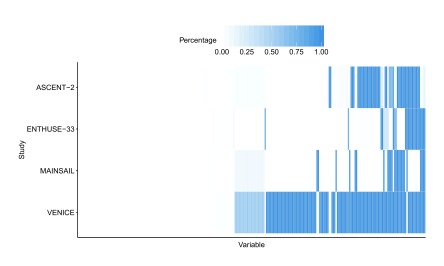
Heatmap of missing value patterns in training and validation data. Darker color indicates higher missing value percentage.

***2.1.3 Multiply imputing covariates***: Missingness was common in the combined dataset. Figure 1 shows the missingness patterns for covariates (columns) within each study (row block). As suggested by the heat map, the missingness is largely study-dependent and likely due to the differences in study protocol and data collection procedure.

The ten continuous covariates with the most missingness are listed in Table 1. Since a considerable proportion of categorical variables were created by categorizing continuous variables (for e.g., labeling lab values as low, normal, or high), these categorical variables have a rate of missingness similar to their continuous counterparts. Other categorical covariates with a large proportion of missingness include a categorization of baseline weight and height (77.6% and 77.3% missing, respectively) and an indicator for a history of smoking (77.1% missing).

**Table 1.** Continuous variables with greatest proportion of missing values.

Variable name	Percent missing
Free prostate specific antigen	78.4
Brain natriuretic peptide	78.3
Mean corpuscular volume	77.6
Mean corpuscular hemoglobin	77.5
Urine protein creatinine ratio	76.1
Creatinine (urine)	75.1
Chloride	74.6
Bicarbonate	74.6
Uric acid	74.6
Creatinine clearance	72.9

Missing covariate data for the combined dataset was then imputed using multiple imputation ^13^ via the fastpmm function in the R package mice (R 3.2.1). Multiple imputation was performed using covariate data from both training (ASCENT-2, VENICE, MAINSAIL) and validation (ENTHUSE-33) studies and was repeated to obtain five completed datasets.

***2.1.4 Covariate standardization***: We standardized continuous data by applying the Box-Cox transformation (with power parameter 0.2) to all continuous covariates, followed by mean-variance standardization.

***2.1.5 Survival summaries***: Figure [X of the main paper] shows the the Kaplan-Meier estimates of survival curves along with the 95% confidence band for each of the three studies in the DREAM Challenge training data. The three studies have similar survival curves up to 17 months from baseline.

## 3 Methods

In this section, we describe an iterative imputation procedure that can be used in tandem with ensemble learning methods to predict survival times given possibly many covariates. This method constitutes our wining algorithm for the Prostate Cancer DREAM Challenge’s sub-challenge **1b** for predicting exact survival times. Throughout our presentation, we use integrated area under the curve (iAUC) to evaluate predictive accuracy and select optimal values for tuning parameters ^19^.

Let ( *Y _i_*_,_Δ _*i*_) be the pair of observed or censored survival times and the censoring indicator for patient *i* = 1, … , *N*. Δ _*i*_ = 1 if *Y _i_* is the observed survival time and 0 if censored. Let **X**_*i*_ be the vector of covariates. We describe our prediction algorithm below in three steps.

I. Initial survival time imputation without covariates
  For individuals with censored survival times– *I*_0_ = { *i* | Δ _*i*_ = 0}–add independent exponential random numbers to the right-censored survival times, i.e., $Y_{i,new}^{\left(0\right)}=Y_{i}+E_{i},$ where *E _i_* ~ Exp( *α*), for *i* ∈ *I*_0_. For individuals with observed survival times, no imputation is necessary; keep the observed *Y _i_*.
  Note that *α* is a tuning parameter for this initial step (as well as throughout the prediction algorithm). We select a value for *α* with a grid search that seeks to maximize the 10-fold cross-validated iAUC. In the initial imputation step, the value of *α* is set to be study-specific but constant across covariates within a study (given exploratory analysis showing heterogeneity across trials). As a result, the values of *α* chosen are: 400 (ASCENT-2), 420 (MAINSAIL), and 460 (VENICE).
II. Adjust imputed survival times using covariates
  We then use covariates to build a predictive model for the completed survival times. Specifically, we iterate between two processes: training an ensemble prediction model (step **IIa**) and adjusting the survival times (step **IIb**) for iterations *k* = 1, … , *K*.
  IIa) Select features and train prediction models:
*Feature selection* Feature selection proceeds using the following three models to identify salient predictors of (log-transformed) survival time: regularized random forest (RRF) with two predictors sampled for splitting at each node (regularization parameter = 0.95); support vector machine (SVM) regression with radial kernel (bandwidth = 0.02, center = 0.15); and, partial least squares (PLS) regression with two components.
  Each model returns a vector of variable importance (VI), which is calculated by R package caret and within the range of 0 – 100. VI vectors are averaged across the three models to obtain a mean VI vector. We then choose "important variables", which we define as those with a final VI greater than tuning parameter *γ* = 24 (chosen to maximize cross-validated iAUC.) Covariates with the highest VI are discussed in section 4.4
*Ensemble model training and predicting *Using selected features, we train five prediction models (listed in Table 2). Tuning parameters for each model were chosen by 10-fold cross-validation to maximize iAUC.

**Table 2.** Models for survival time prediction at imputation step II.

Model name	Tuning parameter	Tuned value at IIa	Tuned value at III	R package
Regularized random forest	mtry, coefReg	2, 0.9	2, 0.9	RRF
SVM-RBF kernel	*σ, C*	0.001, 0.1	0.002, 0.3	e1071
Quantile random forest	mtry	6	6	quantregForest
SVM-Polynomial kernel	degree, scale, C	3, 0.0005, 0.15	5, 0.0005, 0.3	e1071
Partial least square	ncomp	2	2	pls

Trained prediction models are then used to obtain out-of-sample predictions for survival time. In the case of 10-fold cross-validation, covariate and outcome data on 90% of patients are used for training prediction models which then, in turn, provide out-of-sample survival time predictions for the remaining 10% of patients.
  IIb) Adjust imputed survival times:
  For each censored individual (Δ _*i*_ = 0), predicted survival times from each prediction model ( Table 2) are averaged to $Y_{i,adj}^{\left(k\right)},$ where *k* is the iteration number for step **II**. We adjust predicted survival times as follows: $Y_{i,new}^{\left(k\right)}=Y_{i,adj}^{\left(k\right)}$ if $Y_{i,adj}^{\left(k\right)}>Y_{i};$ otherwise, $Y_{i,new}^{\left(k\right)}=Y_{i}+E_{i}^{\left(k\right)},$ where $E_{i}^{\left(k\right)}∼\text{Exp}(\alpha^{*}=80).$ (Here, α* is a tuning parameter whose value is determined by a grid search to maximize the 10-fold cross-validated iAUC.) This adjustment serves to increase under-estimated imputed values to a random quantity greater than the observed censoring time.
  Using these imputed survival times, ensemble survival time prediction (IIa) is repeated. The training and adjustment process is repeated until the incremental increase in cross-validated iAUC is smaller than a pre-set threshold. In our application, we used a relatively large threshold (0.2) to avoid over-fitting, and the algorithm converges after just three iterations.
  More generally, steps **IIa** and **IIb** are repeated several times, say *K*, in order to obtain the adjusted survival imputations $\left\{Y_{i,new}^{\left(K\right)},i\in I_{0}\right\}$ produced by the last iteration. We combine these values with the observed (uncensored) survival times and use them as the complete outcome vector for constructing a final prediction model.
III. Final predictions for patients in the validation dataset
*Individual model.* We trained five prediction models ( Table 2) using log-transformed $Y_{i,new}^{\left(K\right)}$ and Box-Cox transformed features selected in the final ( *K*th) iteration of step **IIa** above. We chose tuning parameters in order to maximize 10-fold cross-validated iAUC; tuned parameter values are listed in Table 2. In this application, we used the same five modeling approaches for both the imputation and prediction steps, though using the same models is not necessary.
*Super learner.* Because we have *I* = 5 multiply-imputed covariate datasets (see *Data cleaning*), the prediction procedure described above can be used to produce distinct sets of survival time predictions for all combinations of *I* = 5 datasets and *M* = 5 survival time prediction models. For each prediction model, we average the resulting out-of-sample (10-fold) predictions for each of the *I* = 5 imputed datasets. Finally, we fit a LASSO regression model with log-transformed survival time as the outcome to determine the optimal weights for combining predicted survival times from the *M* = 5 models. The final output is a predicted survival time based on patient covariate data.

**Figure 2.**
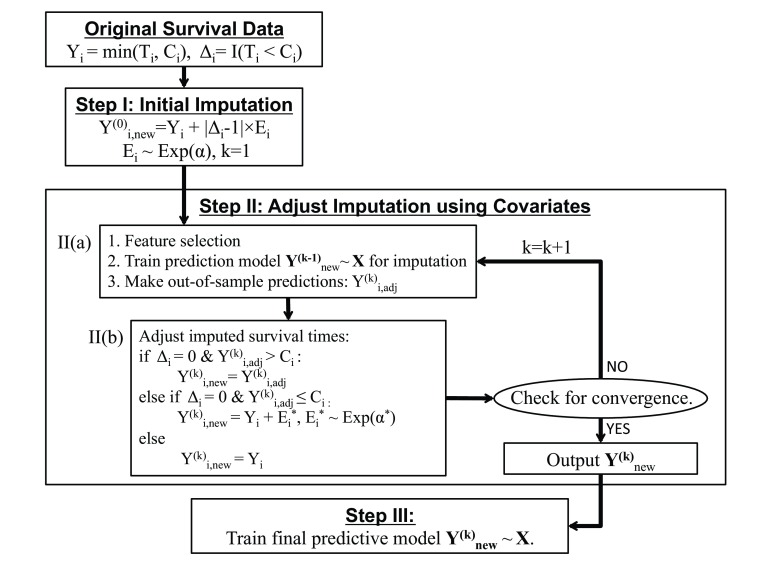
Summary of the imputation and prediction algorithm.

This algorithm is summarized in Figure 2.

## 4 Results

### 4.1 The behavior of iterative imputations

**Figure 3.**
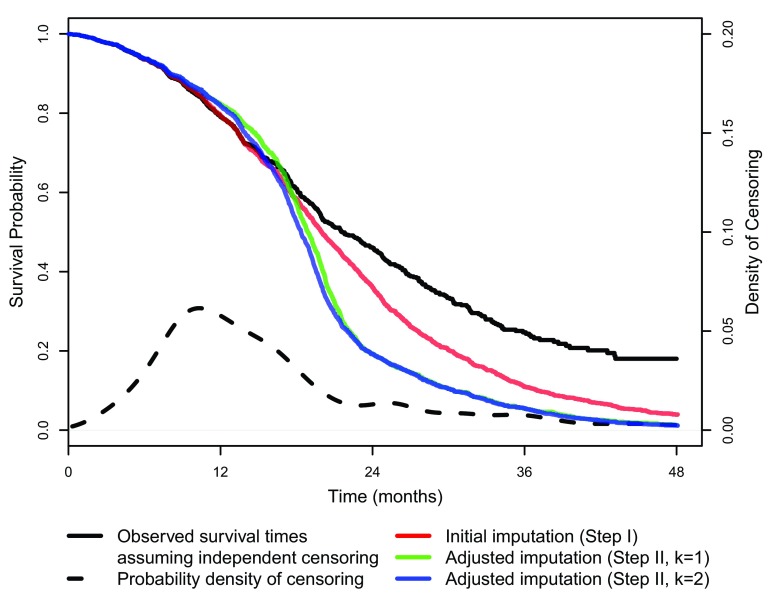
Kaplan-Meier curves of observed and imputed survival times.

Figure 3 displays Kaplan-Meier (KM) estimates for observed survival data and several stages of survival time prediction. The black curve shows the KM estimate for the observed survival data assuming independent censoring. The density function for censoring is given by the dashed black line and indicates that most censoring occurred between six and 20 months.

The red, green, and blue curves show the KM estimates for survival predictions after initial random imputation (step **I**) and *k* = 1 and *k* = 2 iterations of the covariate-based, adjusted survival time predictions (step **II**), respectively. All imputed survival time curves closely track the observed survival curve until 16 months follow-up, at which time survival decreases more rapidly than expected under the assumption of independent censoring.

We note that it is possible for survival estimates after initial imputation (red curve) to lie above or below the observed KM curve (black) depending on larger or smaller choices of *α*, respectively. Here, we see that cross-validation favors larger values of *α* suggesting that censored individuals likely experience shorter-than-average survival after censoring. Survival estimates of model-based predictions (green and blue curves) also suggest that patients censored earlier are expected to have an event around 13–23 months. The green and the blue curves are very similar, indicating that the imputation algorithm converges very quickly.

**Figure 4.**
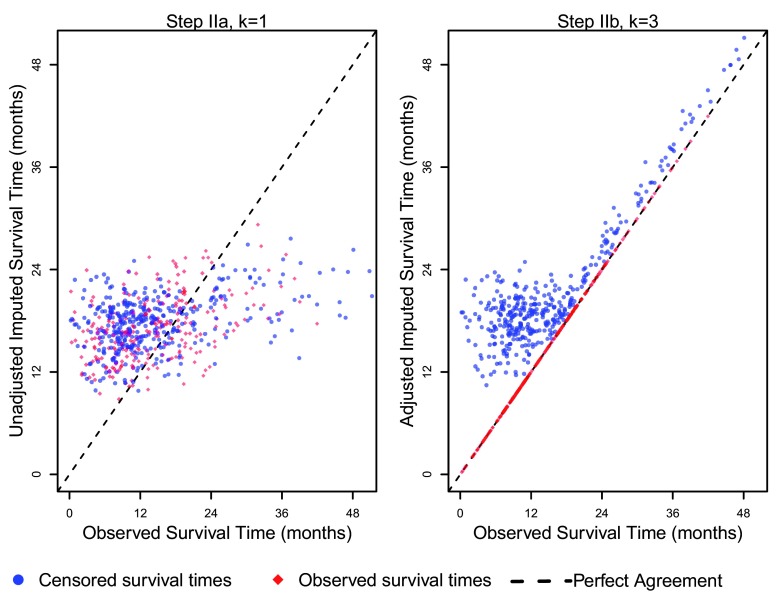
Observed vs. imputed survival times.

The left hand panel of Figure 4 shows a plot of the observed times against the out-of-sample predicted times $Y_{i,adj}^{\left(1\right)}$ made in the first predictive iteration ( *k*=1) in step **IIa**, prior to adjusting prediction in step **IIb**. By the imputation algorithm we proposed, we keep a patient’s survival time *Y _i_*_,__*new*_ = *Y _i_* if an event was observed and censoring did not occur(Δ *_i_* = 1) and impute a patient’s survival time by *Y _i_*_,__*new*_ = *Y _i_* + *E _i_* if *Y _i_*_,__*adj*_ < *Y _i_* and for patients with censored survival time (Δ _*i*_ = 0). The right hand plot shows that, after multiple iterations of this algorithm (k=3), the final imputed values show greater risk stratification for censored patients (blue circles). Because we use observed event times instead of predicted event times for uncensored patients (red diamonds), these observations lie directly on the line of equality (black dashed line).

The left panel in Figure 4 also indicates regression to the mean, i.e., the initial imputations tend to overestimate earlier survival times ( *Y _i_* < 16 months) and underestimate later survival times ( *Y _i_* > 16 months), resulting in a horizontal cloud of points. Our imputation algorithm deals with the underestimation at later survival times by forcing the imputed times to be larger than the observed censoring time, i.e., by the *Y _i_*_,__*new*_ = *Y _i_* + *E _i_* step. On the other hand, overestimation at earlier survival times is controlled by tuning the rate parameters of the exponential distributions ( *α*, α*) in steps **I** and **IIb**. The right panel of Figure 3 shows that the patients with earlier censoring times (circles toward the lower left) have larger differences between the imputed survival time and the observed censoring time ( *y* — *x*) in comparison to patients who survive longer (circles toward the upper right).

### 4.2 Survival time prediction

Although iAUC was used for evaluating the prediction performance in the training stage to make better use of the censored data, root mean squared error (RMSE) based on uncensored observations is used as the scoring metric for survival time prediction accuracy. Based on the training set, the 10-fold cross-validated RMSE of our ensemble predictive model is 246.5. (In the following section, we compare our method with other benchmarks with respect to the cross-validated RMSE using the same data-splitting index.)

In the final scoring round of the DREAM Challenge, our model was trained on the entire training set and then tested on the validation dataset from an independent clinical trial (ENTHUSE-33). Our final ensemble predictive model yielded a RMSE of 198.1 and was one of the top performing algorithms. Our predictions ranked sixth overall in accuracy are were not significantly different from the most accurate survival time predictions (Bayes factor > 3) [placeholder for main challenge paper].

### 4.3 Comparison with benchmark prediction methods

We also compared RMSE of the proposed method to that of an off-the-shelf method: survival random forest (SRF). Ishwaran *et al*. (2008) ^8^ propose a popular SRF method which outputs ensemble cumulative hazard function predictions $\hat{\Lambda }(t|{\text{x}}_{i}),$ enabling one to specify the survival function $\hat{S}(t)=\exp\{-\hat{\Lambda }(t|{\text{x}}_{\text{i}})\}$ for subject *i* = 1, … , *n* at time *t*. We predicted the exact survival time using the *q*% quantile of the estimated survival curve with *q* common to all subjects and selected by 10-fold cross-validation to minimize RMSE. Survival random forest is distinguished from the usual random forest methods by the criterion for choosing and splitting a node. In our implementation, we used a log-rank splitting rule that splits nodes by maximizing the log-rank test statistic ^10, 20^. We increased the speed of training using a *randomized* log-rank splitting rule meaning that, at each splitting step of growing a tree, we randomly split the candidate covariates and choose the covariate and split point pair that maximize the log-rank statistic. This randomized scheme is recommended to avoid overly favoring splitting continuous covariates when both continuous and categorical variables exist.

We generated 1, 000 bootstrap samples from the original training data (compiled and completed as detailed in section 2). We grew one survival tree for each bootstrap sample. The survival random forest produces the final ensemble survival function prediction by averaging over predictions obtained from these trees. To split a node in each tree, we tried a maximum of 10 random splits to determine which variable to split and where to split. Averaged over the five imputed datasets, we obtained a 10-fold cross-validated RMSE 344.8 with *q*% = 37%. Thus, our proposed algorithm performed considerably better (RMSE = 246.5).

### 4.4 Predictors of survival time

Via ensemble prediction modeling, we also identified the most salient predictors of survival time in this population. The strongest predictors of survival time included lab values indicating overall health and cancer activity and other measures of overall health. For example, alkaline phosphate (ALP)– the most predictive covariate–is typically elevated in individuals with metastatic disease. ALP was included as covariate in the Halabi *et al*. (2014) benchmark model ^21^. Other lab measurements in the benchmark model–lactase dehydrogenase (LDH), hemoglobin (HB), prostate specific antigen (PSA), and albumin (ALB)–were also among the most predictive covariates in our model. The Eastern Cooperative Oncology Group (ECOG) performance status (a standard measure of daily living abilities) and use of opiate medication were also included in the Halabi *et al*. (2014) nomogram and were found to be highly predictive of survival in our approach. Disease site, the remaining predictor in the benchmark model, was not among the strongest predictors of survival in our model.

## 5 Discussion

In this paper, we have introduced a survival time prediction method based on multiple imputation and ensemble learning. It is designed for right-censored survival data with many covariates. The proposed method operates by iterating through two stages: iterative imputation of right-censored outcomes and building an ensemble predictive model of survival time. Compared to the existing methods for survival time prediction, the second phase of this algorithm is particularly effective in leveraging covariates to guide imputation of the censored survival times. By imputation, we have transformed the difficult problem of time-to-event prediction with censoring to a standard predictive regression problem. The results of the Prostate Cancer DREAM Challenge 1b have empirically validated the predictive performance of our algorithm. Further research is needed to explore theoretical characteristics of the proposed algorithm. Conceptually, the iterative imputation algorithm achieves strong predictive performance by first generating model-based imputations (which makes use of the covariate information) and, then, correcting survival time predictions based on observed outcomes.

For future work, we will compare our method with other methods such as risk set imputation (RSI) ^14^ and recursively imputed survival trees (RIST) ^22^ using more extensive simulation studies. We will also seek to establish the MSE optimality behind this algorithm and further improve its imputation and prediction performance. In particular, we will further study the impact of the initialization strategy in step I on the final predictive accuracy to explore whether using model-based initialization (such as RIST) performs better than the current cross-validation-based random initialization. Finally, obtaining reliable confidence intervals around predicted survival time is also crucial for this method to be more clinically useful.

## 5.1 Data availability

The Challenge datasets can be accessed at: https://www.projectdatasphere.org/projectdatasphere/html/pcdc Challenge documentation, including the detailed description of the Challenge design, overall results, scoring scripts, and the clinical trials data dictionary can be found at: https://www.synapse.org/ProstateCancerChallenge  The code and documentation underlying the method presented in this paper can be found at: http://dx.doi.org/10.7303/syn4732982 ^31^
