# Supplementary material for: Predicting survival time for metastatic castration resistant prostate cancer: An iterative imputation approach
Source: F1000Res. 2016 Nov 16;5:2672. [Version 1] doi: 10.12688/f1000research.8628.1 (PMC5321124; doi:10.12688/f1000research.8628.1)
Supplement: Peer review history

# Peer review: Referee response for version 1

**DOI:** 10.5256/f1000research.9282.r19040

**Published:** 2017-02-20

**Authors:** C Jason Liang, Wenjuan Gu

**Affiliations:** 1 Biostatistics Research Branch, National Institute of Allergy and Infectious Diseases, Rockville, MD, USA

This article provides a clear summary of how the team's prognostic model was created. Biomedical prognostic models are frequently built with survival data, but in practice often do not fully address or utilize the complexity of the data (e.g. dichotomizing the time-to-event outcome out of convenience rather than scientific motivation), so it was encouraging to read about a method thoughtfully developed for survival data, and a competition that embraces performance measures tailored for survival data. We have no major comments but will provide some minor comments for the authors' consideration.

**Other methods**

 The authors' method involves an initial iterative imputation method that is attractive in that it opens up a richer suite of continuous outcome models for use with the completed data. However, as the authors mention in their discussion, the theoretical aspects of this imputation procedure are unclear. To that end, we are curious if the authors considered existing methods that try to formally account for censoring while retaining loss functions that reduce to "typical" loss functions when censoring is absent (e.g. mean-squared error). For example, Steingrimsson et al (2016) ^1^ and Molinaro et al (2004) ^2^ study random forest models with loss functions that 1) can accommodate censored outcomes; and 2) reduce to squared error loss when censoring is absent.

**Covariate imputation**

 The authors provided a useful graphic summarizing the covariate missingness. Given that there was a nontrivial amount of missingness, a sensitivity analysis might be helpful to ensure that the results are not qualitatively different when perturbing certain aspects of the imputation procedure. Alternatively, for each of the most salient predictors, examining what proportion of observations is missing for that variable may also be useful.

**Requests for clarification**

- The authors mentioned that three datasets were used for training (ASCENT-2, MAINSAIL, and VENICE) and a fourth dataset was used for scoring (ENTHUSE-33). However, in the "data cleaning" section and the "super learner" section there is also reference to five different datasets. It was unclear how to reconcile the two descriptions.
- In Step I of the Methods section, how do you calculate iAUC in the initial cross-validation step to determine \alpha, where no covariates are used? Don't you need a score - presumably derived from the covariates - to calculate iAUC?

**Choice of iAUC and other performance measures by the DREAM challenge organizers**

 We commend the competition organizers for embracing prognostic performance measures that are specifically tailored for survival data, such as the concordance index and cumulative AUC. However, we are puzzled by the decision to use iAUC as the primary performance measure.

 iAUC is not a standard performance measure and, to our knowledge, is not documented in the literature. While this would not necessarily preclude iAUC from being used as the primary performance measure, it would be helpful to understand the justification for its choice. There does not appear to be an immediately obvious interpretation for iAUC. According to the DREAM website, iAUC is the average of the different cumulative AUC values over all times t. While the cumulative AUC for a single t is easily interpretable, it is unclear what the interpretive value of iAUC is.

 Note that Heagerty and Zheng (2005; Section 2.2.1) ^3^ state: "[Cumulative AUC is] most appropriate when a specific time t' (or a small collection of times t'_1, t'_2, ..., t'_m) is important and scientific interest lies in discriminating between subjects who die prior to a given time t' and those that survive beyond t'."

 If one were unable to choose a specific time or small collection of times, the concordance index offers a reasonable "global in time" alternative. Incidentally, Heagerty and Zheng (2005; Section 2.4) ^3^ note that when the incident/dynamic AUC (related to but different than the cumulative AUC) is averaged over time (and subject to a specific time-weighting), the result is the concordance index.

**Typos**

- Section 3, Paragraph 1, Sentence 2: "winning" is misspelled.
- Section 4.4, Sentence 4: "...included as a covariate..."
- Section 4.2, last sentence: "...overall in accuracy and were not..."

We have read this submission. We believe that we have an appropriate level of expertise to confirm that it is of an acceptable scientific standard.

## References

[1] SteingrimssonJADiaoLMolinaroAMStrawdermanRL: Doubly robust survival trees. *Stat Med*.2016;35(20) : 10.1002/sim.6949 3595-612 10.1002/sim.6949 27037609PMC7286558

[2] MolinaroADudoitSvan der LaanM: Tree-based multivariate regression and density estimation with right-censored data. *Journal of Multivariate Analysis*.2004;90(1) : 10.1016/j.jmva.2004.02.003 154-177 10.1016/j.jmva.2004.02.003

[3] HeagertyPJZhengY: Survival model predictive accuracy and ROC curves. *Biometrics*.2005;61(1) : 10.1111/j.0006-341X.2005.030814.x 92-105 10.1111/j.0006-341X.2005.030814.x 15737082

# Peer review: Referee response for version 1

**DOI:** 10.5256/f1000research.9282.r18892

**Published:** 2017-01-09

**Authors:** Ruoqing Zhu

**Affiliations:** 1 Department of Statistics, University of Illinois at Urbana-Champaign, Champaign, IL, USA

The paper is nicely written, and the method is clearly described. 

 I have only one comment regarding the initial imputation step: why an exponential distribution was chosen? Does that affect the results? Can the authors provide a brief discussion on this choice? In the literature, both RSI and RIST uses a model-based imputation value.

I have read this submission. I believe that I have an appropriate level of expertise to confirm that it is of an acceptable scientific standard.

# Peer review: Referee response for version 1

**DOI:** 10.5256/f1000research.9282.r17699

**Published:** 2016-12-07

**Authors:** Devin C. Koestler

**Affiliations:** 1 Department of Biostatistics, University of Kansas Medical Center, Kansas City, KS, USA

In the manuscript entitled, “Predicting survival time for metastatic castration resistant prostate cancer: an iterative imputation approach” Deng and colleagues describe a generalizable algorithm for iteratively imputing event-times for censored observations and apply their methodology to data collected as part of the Prostate Cancer DREAM Challenge. The approach itself is very interesting, and its application within an ensemble-based framework as a means toward informing survival predictions is quite creative. The Introduction provides a nice appraisal of existing methodologies and their limitations, and in the opinion of this reviewer, adequately motivates methodology being proposed. Overall, the manuscript is well written and likely to be of interest to the prediction and machine learning communities. Some suggestions for improvement are given in the space that follows:

 Major comments:

1. Augment the Results section with a table or figure that captures the results generated in the training phase of the authors algorithm, i.e., scatterplot of observed versus predicted survival time based on the 10-fold cross validation procedure or a Bland-Altman plot. It would also be useful to know what features were selected to build the final prediction model that was applied to the validation data set.  Lastly, what were the optimal weights for combining the predicted survival times from the M = 5 models? 

 

 Minor comments:

1. Abstract - “…a recent crowd-sourced competition focused on risk and survival time predictions for patients with…”.  I would be careful about the use of the term “risk” here since the competition did not consist of predicting one’s risk of mCRPC, but rather “risk of early treatment discontinuation”.
2. Abstract – “We are interested in using a patient’s covariates to predict his or her time until death after initiating standard therapy”. I would recommend removing “her” since the study population is men diagnosed with mCRPC. Alternatively, you can just replace “his or her” with “their”.
3. Introduction – “Many state-of-the-art statistical and machine learning tools cannot be directly applied to censored data while most standard methodologies that do allow for censoring assume independence between censoring and survival time; this assumption is frequently inappropriate”. It would be helpful to include reference(s) to support the statement that the assumption of independence of censoring and survival time is inappropriate. In addition, describing the potential inappropriateness of this assumption (and its consequences) in the context of the data set(s) considered here would help further reinforce this point. 
4. Results – “Our predictions ranked sixth overall in accuracy and were not significantly different from the most accurate survival time predictions (Bayes factor > 3)”. My suggestion would be to replace the last part of this sentence with, “…not significantly different from the model that achieved the most accurate survival time predictions (Bayes Factor < 3 compared to the top-ranked model in this subchallenge).
5. Data 2.1.5 Survival Summaries – Might be helpful if you could briefly summarize the censoring rates and median survival times across the 4 clinical trial data sets.
6. Methods – “We then use covariates to build a predictive model for the completed survival times”. I am struggling with the term “completed” here. Do you mean the “imputed” survival times? Perhaps a better way to say this is, “We then used covariates to build a prediction model using the imputed survival times for censored subjects”.
7. Methods 11b) Adjust imputed survival times – For the purposes of clarity it would be helpful to denote the predicted survival times with hat notation.
8. Results – What are the units for the RMSE? Days? In other words, the average difference between observed and predicted survival time based on your methodology was 198.1 days (in the independent ENTHUSE  33 data set)?

I have read this submission. I believe that I have an appropriate level of expertise to confirm that it is of an acceptable scientific standard, however I have significant reservations, as outlined above.
